# Humic Acid Modified by Being Incorporated Into Phosphate Fertilizer Increases Its Potency in Stimulating Maize Growth and Nutrient Absorption

**DOI:** 10.3389/fpls.2022.885156

**Published:** 2022-05-19

**Authors:** Jianyuan Jing, Shuiqin Zhang, Liang Yuan, Yanting Li, Chengrong Chen, Bingqiang Zhao

**Affiliations:** ^1^Key Laboratory of Plant Nutrition and Fertilizer, Ministry of Agriculture and Rural Affairs, Institute of Agricultural Resources and Regional Planning, Chinese Academy of Agricultural Sciences, Beijing, China; ^2^School of Environment and Science, Australian Rivers Institute, Griffith University, Nathan, QLD, Australia

**Keywords:** humic acid-enhanced phosphate fertilizer, humic acid, biological activity, maize, concentration

## Abstract

Humic acid-enhanced phosphate fertilizer (HAP) is widely applied in Chinese agriculture due to its high efficiency. Although the structural composition and physicochemical properties of humic acid (HA) are significantly altered during HAP production, a clear understanding of the mechanisms underlying the biological effects of HA extracted from HAP fertilizer (PHA) on plant growth is still lacking. In the current study, we extracted PHA from HAP and assessed its effects on the dry biomass, phosphorus (P) and nitrogen (N) uptake, and P absorption rate of maize seedlings when supplied at different concentrations (2.5, 5, 10, and 25 mg C L^−1^) in the hydroponic culture. The root vigor, root plasma membrane H^+^-ATPase activity, and root nitrate reductase activity were also determined as the representative indicators of the root capacity for nutrient absorption, and used to clarify the mechanism by which PHA affects the maize growth and nutrient absorption. The results showed that the dry biomass, phosphorus uptake, nitrogen uptake, and average phosphorus absorption rates were significantly higher by 14.7–27.9%, 9.6–35.1%, 17.9–22.4%, and 22.1–31.0%, respectively, in plants treated with 2.5–5 mg C L^−1^ PHA compared to untreated controls. Application of 10–25 mg C L^−1^ raw HA resulted in similar stimulatory effects on plant growth and nutrient absorption. However, higher levels of PHA (10–25 mg C L^−1^) negatively impacted these indicators of plant growth. Furthermore, low PHA or high raw HA concentrations similarly improved root vigor and root plasma membrane H^+^-ATPase and nitrate reductase (NR) activities. These results indicate that lower concentrations of PHA can stimulate maize seedling growth and nutrient absorption to an extent that is comparable to the effect of higher concentrations of raw HA. Thus, the proportion of HA incorporated into HAP could be lower than the theoretical amount estimated through assays evaluating the biological effects of raw HA.

## Introduction

Field application of humic acid-enhanced phosphate fertilizer (HAP) has been shown to increase crop yields and phosphorus (P) use efficiency when compared to conventional phosphate fertilizers (Li et al., 2017; Ma et al., 2019). Its higher performance is due to the effects of humic acid (HA) on P bioavailability, stimulation of plant growth, and nutrient absorption (Eyheraguibel et al., 2008; Yang et al., 2019; De Hita et al., 2020). However, only trace amounts of HA are present in HAP (i.e., typically lower than 0.5% *w*/*w*) (Zhao et al., 2020), suggesting that HA provides a greater contribution toward stimulating root growth and nutrient absorption than toward modulating P bioavailability (Eyheraguibel et al., 2008; Rose et al., 2014). Thus, the in-depth study of the bioactive effects of HA in HAP (PHA) on the production of plants cultivated in hydroponics is beneficial to clarify the mechanism that HA enhances the efficiency of phosphate fertilizer, and provides an important reference for HAP production (Urrutia et al., 2013).

Many studies have suggested that HA shows strong biological activity in promoting plant growth and the absorption of macronutrients, such as nitrogen (N) and P (Canellas et al., 2010; Jannin et al., 2012; Shah et al., 2018; De Hita et al., 2020; Jindo et al., 2020), and that these effects can be modulated by variations in the structural composition, molecular weight, and concentrations of different compounds in heterogenous HA. For example, HA that is structurally enriched in carboxyl groups and hydrophobic structures has been reported to increase the root surface area, whereas HA with a greater proportion of aromatic and carbonyl groups have been linked to the increase of root number and diameter (García et al., 2016). Alternatively, HA composed of more smaller molecular components is reportedly most effective for inducing the absorption, transport, and assimilation of ${\text{NO}}_{3}^{-}$ (Albuzio et al., 1986; Nardi et al., 2000; Zanin et al., 2018; Pizzeghello et al., 2020), while HA containing a higher proportion of high molecular components has been described as a potent positive regulator of root growth in other studies (Zandonadi et al., 2007; Canellas et al., 2009). Thus, the chemical structure of HA affects its function as a plant growth stimulator, while it is uncertain which chemical structure is predominantly available.

It is well-established that high temperature can substantially alter the structural composition of HA (Zhou et al., 2019), while a lot of heat is generated by exothermic neutralization reactions between phosphoric acid and alkaline compounds during the production of phosphate fertilizers (Peng and Xiang, 2017). Hence, the incorporation of HA into the phosphate fertilizer can change its structure, which has been verified by our previous study (Jing et al., 2020). The change likely leads to differences in the effects and potency of PHA on plant growth compared to carbon equivalent concentrations of HA. Whether PHA also maintained the bioactive effects on plant growth as HA was of great significance for revealing the enhancement mechanism of PHA on the efficiency of phosphate fertilizers.

Additionally, HA concentration can also serve as an important factor that determines its effect on the stimulation of plant growth. Pizzeghello et al. (2020) identified a positive, linear, and concentration-dependent relationship between biomass production and HA concentration (ranging from 0 to 1 mg C L^−1^) in garlic. However, work by Rose et al. (2014) indicated that plant biomass production followed a bell-shaped distribution with increasing HA concentration. Likewise, Garcia et al. (2016) observed adverse effects on the root of *Brachiaria* following the application of high concentrations of HA, which can be attributed to redox imbalance. Most of the previous studies focused on the influence of individual factors while overlooking the comprehensive contributions of structural properties, the molecular weight of constituent compounds, and concentration in the mechanistic analyses of the HA effects on plant growth.

The objective of this study is to determine the biological activity of PHA, identify the possible mechanisms by which HAP enhances plant growth and nutrient absorption, and provide a reference for the amount of HA incorporated into phosphate fertilizer. To this end, we extracted PHA from HAP and conducted a series of hydroponic experiments to assess the effects of 2.5–25 mg C L^−1^ of HA or PHA on biomass production, P and N uptake, and P absorption rate in maize. We also examine representative indicators of the root capacity for nutrient absorption, including root vigor and root plasma membrane H^+^-ATPase activity, as well as root nitrate reductase activity as an indicator of nitrogen metabolism for biomass production.

## Materials and Methods

### Preparation of HA and PHA

Humic acid was extracted from weathered coal (45°23′ N, 119°15′ E; Huolinhe, Tongliao, Inner Mongolia Autonomous Region, Northeast China) according to the method conducted by Zhang et al. (2017). Subsequently, HAP incorporated with 0.5% HA was manufactured, and PHA was extracted from HAP and purified as described by Jing et al. (2020). In brief, 56.03 g of potassium hydroxide was added to the mixture containing 0.50 g of HA and 46.47 g of phosphoric acid (85% in *v*/*v*) under continuous stirring, and the reaction product was immediately pulverized and grounded through a 0.85 mm sieve to obtain the HAP fertilizer. Ten samples were prepared to achieve enough amounts of HAP for the extraction of PHA. HAP was dissolved in deionized water at a solid–liquid ratio of 1:10, and the pH of the mixture was adjusted to 1.0 using 6 M HCl. After standing for 24 h, the solution was centrifuged. The insoluble portions were collected and thoroughly washed four times with deionized water at a solid–liquid ratio of 1:10 and then oven-dried at 50°C to obtain PHA.

The phosphorus content of HA and PHA was determined using inductively coupled plasma emission spectrometer (5110 ICP-OES, Agilent Technologies Inc., USA) after wet digestion with H_2_SO_4_-H_2_O_2_ (Kalra, 1998). The contents of carbon, hydrogen, oxygen, nitrogen, and sulfur in HA and PHA were determined using an element analyzer (Vario Micro Cube, Elementar Analysensysteme GmbH, Germany). The relative proportions of C-containing functional groups and the molecular weight distributions of HA and PHA were determined using solid-state ^13^C nuclear magnetic resonance spectrometer (Bruker AVANCE III HD 400 MHz, Switzerland) and gel permeation chromatography (GPC, Shimazu LC-20A, Japan) as described by Jing et al. (2020). The data of the above parameters are presented in Table 1 and Supplementary Figure S1.

**Table 1:** Elemental composition, relative proportions of C-containing functional groups, hydrophobicity index, and the percentage of the fractions with molecular weight <3,500 Da in all the detected molecular weights of HA and PHA.

**Sample**	**Elemental composition (%)**						**Relative proportions of C-containing functional groups (%)**							**HI**	**The percentage of molecular weight ≤3,500 Da (%)**
	**P**	**C**	**H**	**O**	**N**	**S**	**Carbonyl (220–190)**	**Carboxylic acid (190–160)**	**O-Aryl (160–140)**	**C-Aryl (140–110)**	**O-Alkyl (110–60)**	**O-CH_**3**_ (60–45)**	**Alkyl (45–0)**		
HA	0.9	59.5	2.7	31.1	2.5	0.8	0.0	9.4	5.0	81.2	4.0	0.3	0.2	6.4	56.9
PHA	1.1	48.2	3.0	26.1	1.4	0.4	0.1	7.0	6.1	82.1	4.2	0.4	0.2	7.6	75.7

*Hydrophobicity index (HI) = Σ[(0–45 ppm) + (110–160 ppm)]/Σ[(45–60 ppm) + (60–110 ppm) + (160–190 ppm) + (190–220 ppm)], calculated as Bento et al. (2020)*.

### Hydroponic Maize and Experimental Design

Maize hybrid ZD958, a dominant high-yield hybrid in North China, was used in this study. Hydroponic experiments on maize seedling growth were conducted in an artificial climate chamber (28°C day/21°C night, 16 h/8 h light/dark period, 300 μmol m^−2^ s^−1^ light intensity, and 70% relative humidity) at the Dezhou experimental station, Chinese Academy of Agricultural Sciences. Before hydroponic culture, maize seeds were presoaked and pregerminated as described by Jing et al. (2020), and then the endosperm of seedlings was removed, and each plant was transplanted to containers filled with Hoagland nutrient solution (pH = 6.1) (Mao and Shen, 2011) containing HA or PHA at different concentrations. According to the previous studies by our group and others (Jing et al., 2020; Rosa et al., 2021), the concentration gradient of HA or PHA was set as 2.5, 5, 10, and 25 mg C L^−1^. The nutrient solution with 0 mg C L^−1^ of HA or PHA was set as a control. Six replicates were arranged for each treatment. During the incubation period, the nutrient solution was renewed every 72 h.

### Sampling and Laboratory Analyses

During the incubation, the volume of initial and replaced nutrient solution was accurately measured, and meanwhile, the sampling of nutrient solution was conducted for the further P concentration analysis. The P concentration in the sampled nutrient solution was determined by the vanadium molybdate yellow colorimetric method (Haslemore and Roughan, 1976). Then, the P absorption rate of the plant was calculated according to formula (1).

On the 30th day after seed germination, the plant was harvested and divided into roots and shoots. All replicates for each treatment were split into two halves. One-half was stored in liquid nitrogen to retain freshness, and the remaining half was oven-dried at 105°C for 30 min and then at 75°C for 48 h to determine the dry biomass of the plants.

The fresh samples were used to measure the physiological traits as follows. The root vigor was evaluated by TTC (2, 3, 5-triphenyltetrazolium chloride) reduction method (Chen et al., 2006). The root plasma membrane (PM) H^+^-ATPase activity was determined by using Plant H^+^-ATP ELISA Kit (Jianglai Biological, Shanghai, China). The root nitrate reductase (NR) activity was assayed by monitoring the nitrite formation by the colorimetric method, as indicated by Jaworski (1971). The oven-dried samples were ground using a ball mill (MM400, RETSCH, Germany), and the P and N content was determined after wet digestion with H_2_SO_4_-H_2_O_2_ (Kalra, 1998). The P content was determined using the vanadium molybdate yellow colorimetric method (Johnson and Ulrich, 1959), and the N content was determined using the Kjeldahl method (Bremner, 1960).

### Calculations and Statistical Analysis

Based on the decrease in the amount of P in the nutrient solution per unit time, the P absorption rates (mg day^−1^) of a plant can be calculated as:


(1) $$\begin{array}{l} P\;absorption\;rate=\frac{C_{1}\times V_{1}-C_{2}\times V_{2}}{D} \end{array}$$

where C_1_ and C_2_ are the concentrations of P (mg/ml) in the initial and replacement of nutrient solution, respectively; V_1_ and V_2_ are the volumes of initial and replacement of nutrient solution (ml), respectively; and *D* is the number of days that the nutrient solution was used, and D was 3 days in this study.

All values are shown as the mean of all replicates. The variance among different treatments was analyzed by using SAS 9.1 (SAS Institute Inc., NC, USA). The differences in maize traits between HA and PHA treatments at the same concentration were compared with two independent sample *t*-tests, while the differences between different HA or PHA concentrations were evaluated by performing an analysis of variance (ANOVA) with the least significant difference (LSD) (α = 0.05). LSD was also used to assess the interaction between the type and concentration of HA and PHA. We conducted a structural equation model (SEM) to explore the direct and total effects of root vigor, and root PM H^+^-ATPase and root NR activities on the nutrient absorption, and Pearson correlation analysis was used to explore the relationship between nutrient absorption and root vigor or root PM H^+^-ATPase activity. Graphs were compiled by using Origin 2021 (Origin Lab Corporation, MA, USA).

## Results

### PHA Shows Stronger Stimulatory Effects on Maize Biomass Production Than HA at Low Concentrations

**Figure 1:**
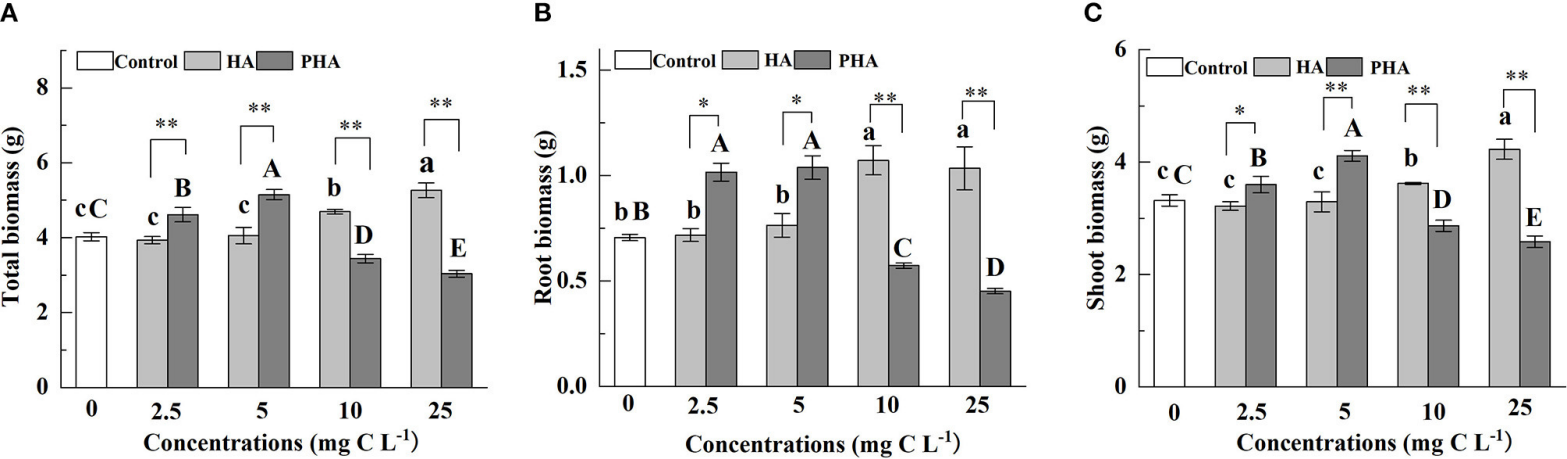
Effects of 2.5–25 mg C L^−1^ of HA or PHA on the **(A)** total dry biomass, **(B)** root biomass, and **(C)** shoot biomass. Error bars represent standard deviations (*n* = 3). Different lowercase letters above columns indicate significant differences between HA and control treatments, while different capital letters above columns indicate significant differences between PHA and control treatments at *P* < 0.05, as determined by the LSD test. Significant differences between HA and PHA treatments at equivalent carbon concentrations were compared with two independent sample *t*-tests, *P < 0.05; **P < 0.01.

To better understand the effects of PHA on the growth of maize plants, we first compared biomass production between maize plants treated with different concentrations of HA or PHA. The results showed that humic acid type, the concentration, and the interaction between type and concentration significantly impacted the accumulation of maize dry biomass (*P* < 0.01) (Supplementary Table S1). HA led to 16.6% and 30.7% higher total dry biomass than control plants at 10 and 25 mg C L^−1^ (*P* < 0.05), respectively, although it had no significant impact on total dry biomass at the concentrations of 2.5 or 5 mg C L^−1^ (Figure 1A). In contrast, application of 2.5 or 5 mg C L^−1^ PHA significantly increased total dry biomass by 14.7 and 27.9%, respectively, compared to the untreated controls (*P* < 0.05), but resulted in a significant decrease in biomass of 14.5 and 24.6% at 10 and 25 mg C L^−1^ concentrations, respectively (*P* < 0.05). A comparison of HA and PHA at the equivalent carbon concentration showed that biomass production was significantly higher under PHA treatment at low concentrations (i.e., 2.5–5 mg C L^−1^) (*P* < 0.05), whereas HA application provided significantly stronger effects than PHA at higher concentrations (i.e., 10-25 mg C L^−1^) (Figure 1A). In addition, quantification of root biomass and shoot biomass under HA or PHA treatments recapitulated the effects of different concentrations on total dry biomass (Figures 1B,C). Taken together, these results indicated that PHA provided stronger stimulatory effects on biomass production in maize at lower concentrations than HA.

### PHA Provides Its Maximal Effects on Nutrient Uptake at Lower Concentrations Than HA

**Figure 2:**
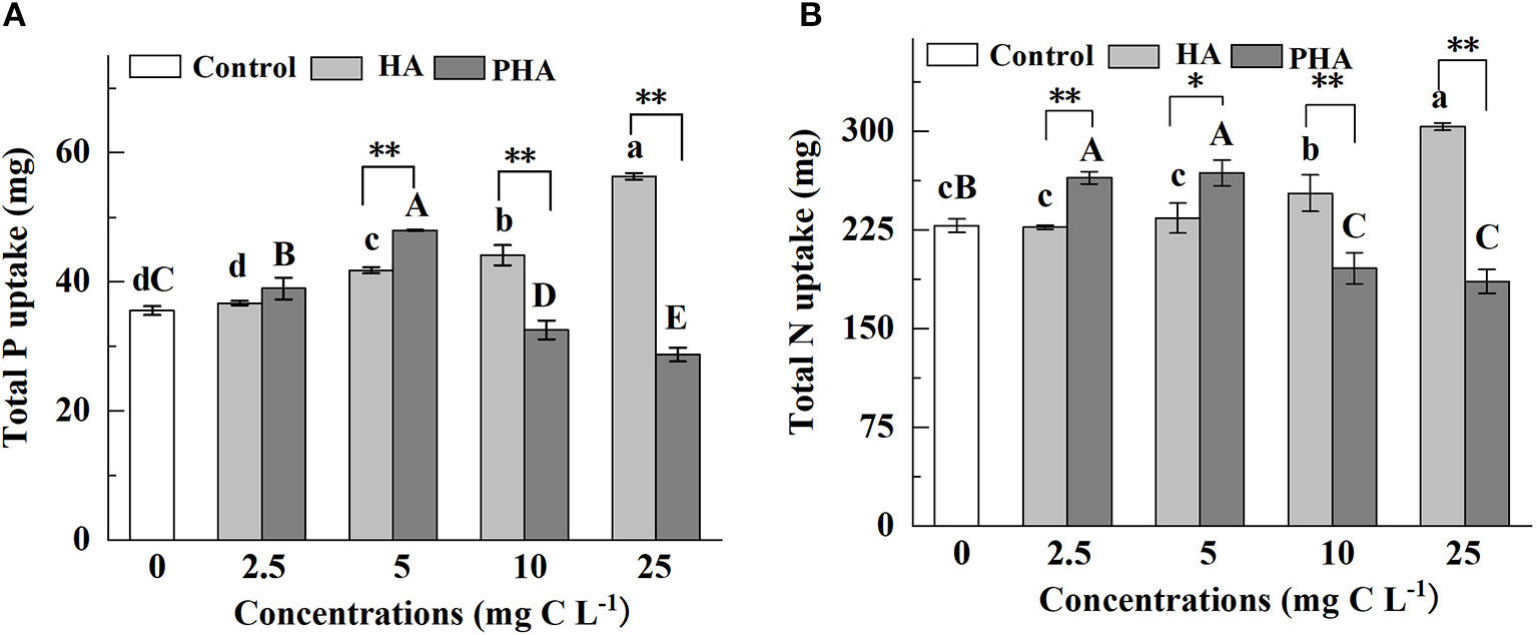
Effects of 2.5–25 mg C L^−1^ of HA and PHA on the **(A)** total P uptake and **(B)** N uptake. Error bars represent standard deviations (*n* = 3). Different lowercase letters above the column indicate significant differences between HA and control treatments, while different capital letters above the column indicate significant differences between PHA and control treatments at *P* < 0.05, as determined by the LSD test. Significant differences between HA and PHA treatments at equivalent carbon concentrations were compared with two independent sample *t*-tests, *P < 0.05; **P < 0.01.

Given that PHA application led to increased biomass accumulation in both above- and below-ground plant organs, we next measured P and N uptake under exposure to different concentrations of PHA or HA (Figures 2A,B). The results indicated that humic acid type, concentration, and the interaction between type and concentration significantly impacted P or N uptake (*P* < 0.01) (Supplementary Table S1). Although HA had no significant impact on P or N uptake at low concentrations (i.e., 2.5–5 mg C L^−1^) (*P* > 0.05), at high concentrations of HA (i.e., 10–25 mg C L^−1^), the uptake of both macronutrients was significantly greater than that observed in the untreated control plants (*P* < 0.05) (Figures 2A,B). Conversely, PHA treatments significantly enhanced nutrient uptake at lower concentrations (i.e., 2.5–5 mg C L^−1^) (*P* < 0.05), but significantly reduced P and N uptake at higher concentrations (*P* < 0.05) relative to that observed in controls (Figures 2A,B). Maximum nutrient uptake for each treatment was observed at 25 mg C L^−1^ HA and 5 mg C L^−1^ PHA, with 58.5 and 35.1% higher P uptake corresponding to these respective treatments (Figure 2A), and 32.9 and 17.5% higher N uptake, respectively, compared to that of controls (Figure 2B).

At equivalent carbon concentrations, nutrient uptake was significantly higher under PHA treatments than that under HA treatment at low concentrations (*P* < 0.05), although no significant differences were identified between HA and PHA treatments in P uptake at 2.5 mg C L^−1^ (*P* > 0.05). At high concentrations (10–25 mg C L^−1^), nutrient uptake was significantly higher in plants treated with HA compared to those subjected to PHA treatment (*P* < 0.05) (Figures 2A,B). Moreover, nutrient uptake by roots and shoots under different concentrations of HA or PHA was generally consistent with total P or N uptake (Supplementary Figures S2A–D).

**Figure 3:**
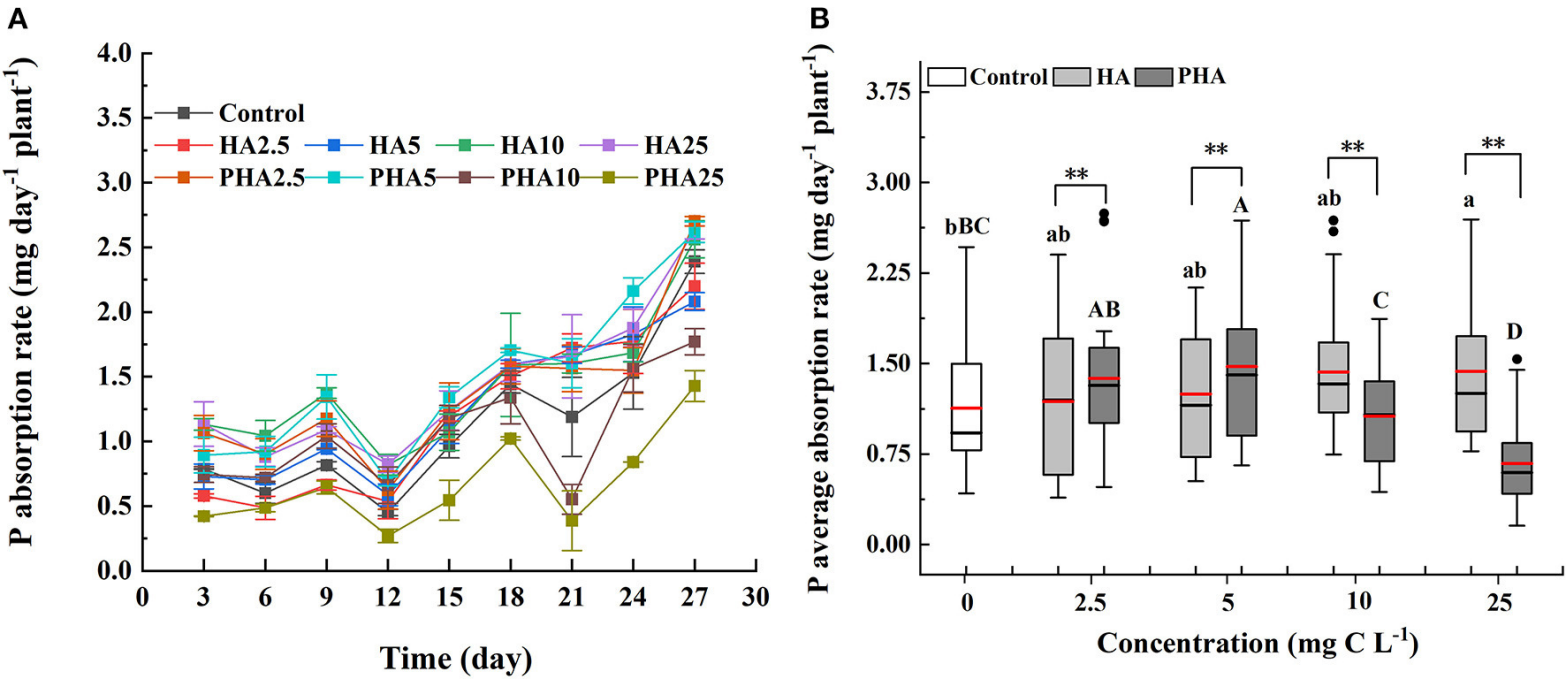
Effects of 2.5–25 mg C L^−1^ of HA and PHA on **(A)** P absorption rate over time (*n* = 3) and **(B)** average P absorption rate (*n* = 27). **(B)** Black lines, median value; red lines, mean value; lower and upper edges of the boxes, 25th and 75th percentiles of all data; bars, 5th and 95th percentiles of all data; dots in outside the boxes, <5th and >95th percentiles of all data. Different lowercase letters above the column indicate significant differences between HA and control treatments, while different capital letters above the column indicate significant differences between PHA and control treatments at *P* < 0.05, as determined by the LSD test. Significant differences between HA and PHA treatments at equivalent carbon concentrations were compared with two independent sample *t*-tests, **P < 0.01.

We then compared the rates of P absorption by maize under different concentrations of HA or PHA over 30 days of cultivation (Figure 3A). During the cultivation, the average rates of P absorption increased along with HA concentration by 4.9, 10.5, 26.7, and 27.2% at 2.5, 5, 10, and 25 mg C L^−1^, respectively, compared to that in the untreated controls (Figure 3B). In contrast, average P absorption rates increased by 21.9 and 30.6% over that in controls under treatment with 2.5 and 5 mg C L^−1^ PHA, respectively. However, average P absorption rates were lower than the rates observed in control plants, by 5.8 and 40.5% on average, under 10 and 25 mg C L^−1^ PHA, respectively (Figure 3B). Comparison of treatments at equivalent carbon concentrations indicated that average P absorption rates by maize were significantly higher in PHA than HA at 2.5 or 5 mg C L^−1^, but significantly higher in HA compared to PHA at 10 or 25 mg C L^−1^ (*P* < 0.05). These results suggested that the positive effects of PHA on nutrient uptake were strongest at lower concentrations than those conferred by HA treatment.

### PHA Provides Maximal Stimulatory Effects on Root Nutrient Absorption Capacity at Lower Concentrations Than HA

**Figure 4:**
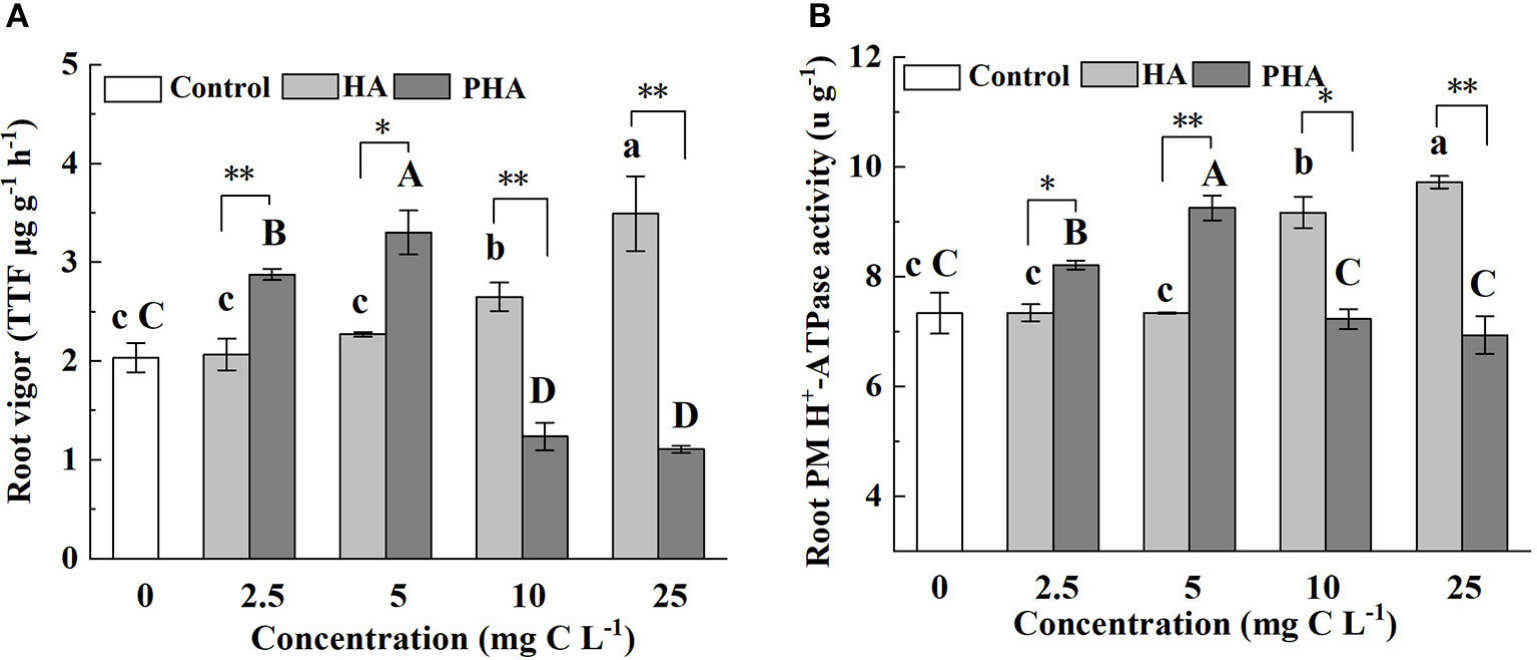
Effects of 2.5–25 mg C L^−1^ of HA and PHA on **(A)** root vigor and **(B)** root plasma membrane (PM) H^+^-ATPase activity. Different lowercase letters above the column indicate significant differences between HA and control treatments, while different capital letters above the column indicate significant differences between PHA and control treatments at *P* < 0.05, as determined by the LSD test. Significant differences between HA and PHA treatments at equivalent carbon concentrations were compared with two independent sample *t-*tests, *P < 0.05; **P < 0.01.

Since root vigor and root plasma membrane (PM) H^+^-ATPase activity are known as representative indicators related to nutrient absorption by roots (Azevedo et al., 2019; Huang et al., 2021), we measured these traits in order to assess the effects of PHA on the capacity of root to absorb nutrients. The results showed that HA treatments resulted in significantly increased root vigor and root PM H^+^-ATPase activity at concentrations of 10 or 25 mg C L^−1^ compared to the control treatments, but had no significant impact on these indicators at 2.5 or 5 mg C L^−1^ (*P* > 0.05) (Figures 4A,B). In agreement with our experimental results, both indicators were significantly higher at 2.5–5 mg C L^−1^ of PHA than that in controls (*P* < 0.05) (Figures 4A,B). However, at 10–25 mg C L^−1^ of PHA, root vigor was significantly lower than that of control plants (*P* < 0.05) (Figure 4A), and root PM H^+^-ATPase activity was not significantly different from controls (Figure 4B). Notably, at 2.5 or 5 mg C L^−1^, root vigor and root PM H^+^-ATPase activity were significantly higher in PHA treatments than in HA treatments, but significantly higher in HA than PHA at 10 or 25 mg C L^−1^ (*P* < 0.05). These results suggested that both PHA and HA application could lead to higher root vigor and root PM H^+^-ATPase activity than that observed in the absence of either treatment, but with maximal effects conferred at lower concentrations of PHA compared to HA.

### Concentration of PHA With Largest Stimulation on Root Nitrate Reductase Is Lower Than That of HA

**Figure 5:**
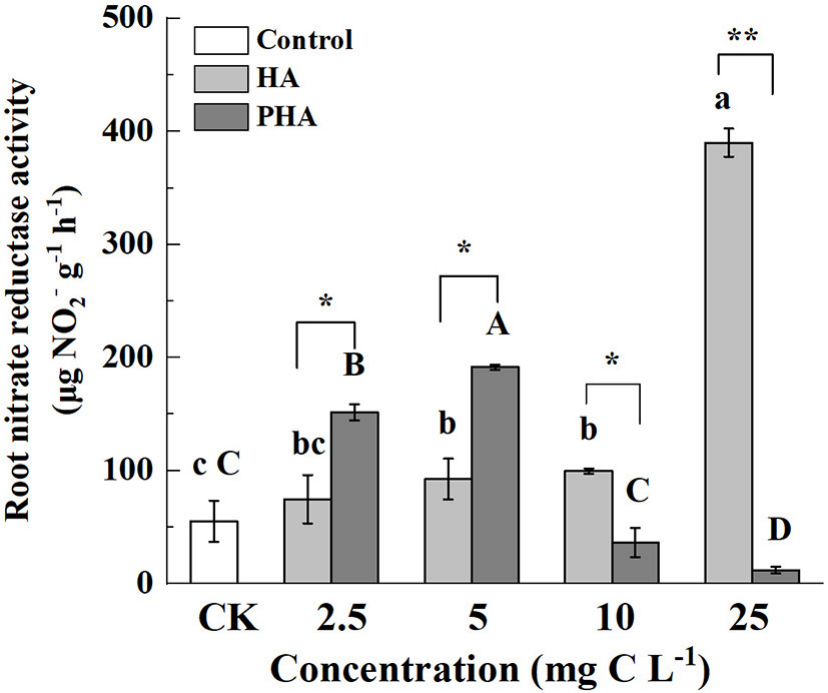
Effects of 2.5–25 mg C L^−1^ of HA and PHA on root nitrate reductase activity. Different lowercase letters above the column indicate significant differences between HA and control treatments, while different capital letters above the column indicate significant differences between PHA and control treatments at *P* < 0.05, as determined by the LSD test. Significant differences between HA and PHA treatments at equivalent carbon concentrations were compared with two independent sample *t*-tests, *P < 0.05; **P < 0.01.

Since nitrate reductase (NR) activity in roots is an important indicator to assess nitrogen metabolism, we investigated whether and how different concentrations of HA or PHA affected this activity. The results showed that root NR activity was significantly higher than that in control plants at high concentrations of HA (i.e., 10–25 mg C L^−1^) (*P* < 0.05), but not significantly different from controls at 2.5 mg C L^−1^ (*P* > 0.05) (Figure 5). However, compared to the NR activity detected in the untreated control plants, the application of 2.5–5 mg C L^−1^ of PHA resulted in significantly elevated root NR activity, while higher concentrations (10–25 mg C L^−1^) of HA significantly decreased root NR activity (*P* < 0.05) (Figure 5). As evident in the above-mentioned comparisons between equivalent treatments, root NR activity was significantly higher in PHA treatments than in HA treatments at low concentrations, but higher in HA treatments than PHA at high concentrations (*P* < 0.05). These results indicated that both PHA and HA could increase root NR activity at different concentrations.

## Discussion

### Modification of Humic Acid *via* Its Incorporation Into Phosphate Fertilizer Lowers the Concentration Required to Stimulate Maize Growth

In this study, we show that the stimulatory effects on plant growth and nutrient absorption by HA or PHA are affected by their concentration (Supplementary Table S1), and the concentration of PHA required to significantly increase dry biomass and nutrient absorption is lower than that of HA, which could be explained by differences in the structural characteristics of HA and PHA. Compared with HA, PHA was characterized by the presence of more low-molecular-weight compounds and a higher hydrophobicity index, and less number of carboxylic groups (Table 1). Previous reports showed that HA with a larger proportion of low-molecular-weight compounds exhibits higher potency in promoting the growth of garlic (*Allium sativum* L.) plantlets than HA containing a larger proportion of high-molecular-weight compounds at concentrations ranging from 0 to 5 mg C L^−1^ (Pizzeghello et al., 2020). Similarly, HA with strong hydrophobicity and more carboxylic groups shows greater beneficial effects on root growth, root area, root mitotic site, and root PM H^+^-ATPase activity (Jindo et al., 2012) at lower concentrations than HA with low hydrophobicity, which requires higher application rates to achieve the same results on plant productivity (Monda et al., 2018). These findings are supported by our results that show lower concentrations of PHA can provide similar stimulatory effects on plant growth as higher concentrations of HA (Figures 1A, 2A,B), which can be attributed to PHA containing a greater percentage of low-molecular-weight components than HA (Table 1, Supplementary Figures S1C,D), while incongruity with the fact that PHA has less carboxyl groups than HA. The result is also inconsistent with the view that HA-mediated promotion of root growth seems to be more closely related to HA mobility, molecular conformation, and functional group distribution than to molecular weight (Olaetxea et al., 2018). The above incongruity might be attributed to the fact that there was a greater variation of the molecular weight distribution between PHA and HA than that of the carboxyl group content (Table 1). Additionally, the result of this research also suggests that the actual quantity of HA, added during the preparation of HAP, that is required to obtain optimal effects on plant growth could be less than that determined by theoretical calculations based on the biological effects of raw HA.

### PHA Can Improve Root Vigor and Root Nitrate Reductase Activity to Enhance Absorption of Macronutrients

**Figure 6:**
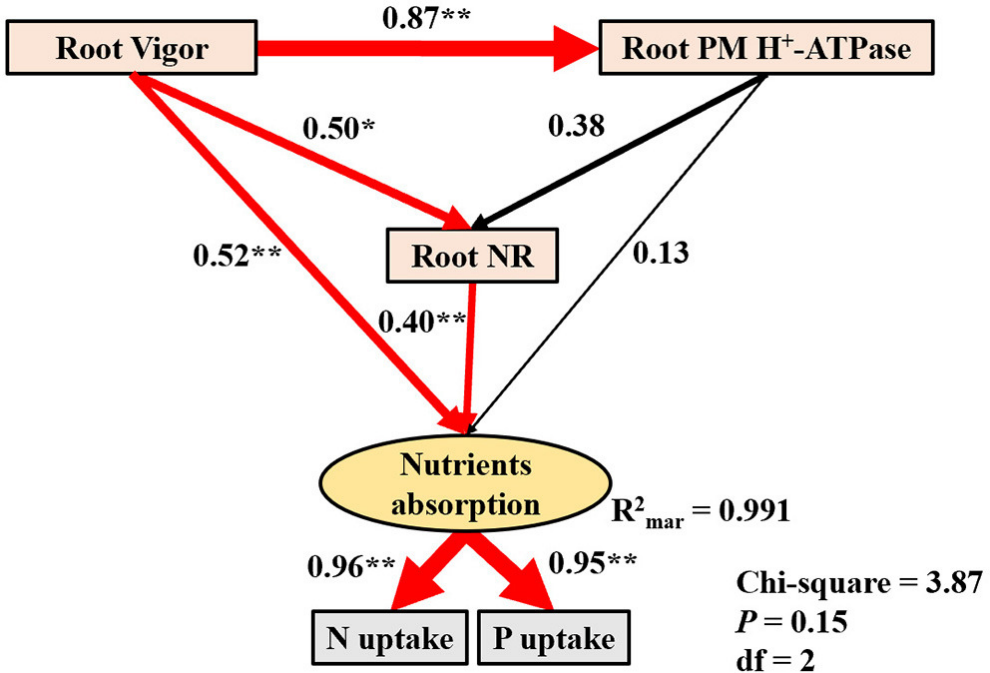
A structural equation model to test the effects of root vigor, root PM H^+^-ATPase, and root nitrate reductase (NR) on nitrogen and phosphorus absorption. The width of the connections represents estimates of the standardized path coefficients, with solid lines representing a positive relationship and dashed lines a negative relationship. Significant connections are shown in red and non-significant connections in black. Significance was determined at: *P < 0.05, **P < 0.01.

The increase in nutrient uptake is usually attributed to the enhancement of root physiological indexes, such as root vigor, root PM H^+^-ATPase activity, and root nitrate reductase (NR) activity (Olaetxea et al., 2018; Azevedo et al., 2019; Zhou et al., 2022), while the primary factor varied for different environmental conditions. Therefore, we constructed a structural equation model to analyze inherent relationships between nutrient uptake and root physiological traits affected by HA or PHA. Our model showed that nutrient absorption (P or N) is directly positively influenced by root vigor (Figure 6), which indicated that PHA application enhances nutrient absorption through improved root vigor when compared to HA.

The higher absorption of P or N in PHA is also directly stimulated by root NR activity (Figure 6), since root NR catalyzes the first step in the reduction of nitrate N to organic forms in plants, and increasing NR activity promotes N accumulation (Cordeiro et al., 2011). This is consistent with previous results that higher root NR activity and more nutrient uptake were observed in maize treated with low-molecular-weight HA (Vaccaro et al., 2015; Zanin et al., 2018). Moreover, root NR activity also has positive effects on P absorption (Figure 6), implying that enzymes associated with N metabolism also contribute to P uptake. This co-regulatory effect on N and P uptake could be explained by the requirement of both essential macronutrients in plant biomass production, for example, nucleic acid and nucleoprotein synthesis (Marschner, 2012). Overall, PHA can improve P or N uptake by enhancing root vigor and stimulating root NR activity.

In addition, previous studies have also reported that the positive effect of HA on root PM H^+^-ATPase activity is the main factor affecting nutrient absorption (Xu et al., 2012; Olaetxea et al., 2018; Azevedo et al., 2019). However, in this study, root PM H^+^-ATPase activity had no significant impact on nutrient absorption (*P* > 0.05) (Figure 6). Further correlation analysis revealed that P or N uptake shares a generally positive correlation with root vigor and root PM H^+^-ATPase activity (*P* < 0.001, *n* = 27), although the correlation coefficients for uptake and root vigor (P uptake, *R*^2^ = 0.90; N uptake, *R*^2^ = 0.94) are higher than those observed for uptake and root PM H^+^-ATPase activity (P uptake, *R*^2^ = 0.88; N uptake, *R*^2^ = 0.85), implying that nutrient uptake is more strongly affected by root vigor than PM H^+^-ATPase activity. This result is supported by the lack of significant effects on nutrient absorption by root PM H^+^-ATPase activity in our structural equation model.

## Conclusion

This study has clearly demonstrated that PHA retains the bioactive effects of raw HA in stimulating maize seedling growth and P or N absorption. HA or PHA appears to increase nutrient absorption by enhancing root vigor and stimulating root NR activity. However, lower concentrations of PHA can provide similar stimulatory effects on plant growth and nutrient absorption as higher concentrations of HA. These results have indicated that the actual proportion of HA being incorporated into HAP for optimal effects on maize growth and macronutrient uptake could be lower than that determined through the assays that evaluated the biological effects of raw HA.

## Data Availability Statement

The raw data supporting the conclusions of this article will be made available by the authors, without undue reservation.

## Author Contributions

BZ conceived and designed the study. JJ and SZ conducted the experiments and wrote the main manuscript. LY, YL, and CC contributed to data interpretation. All authors were involved in revising the manuscript.

## Funding

This work was financially supported by the National Key Technologies R&D Program of China during the 13th Five-Year Plan period (2016YFD0200402).

## Conflict of Interest

The authors declare that the research was conducted in the absence of any commercial or financial relationships that could be construed as a potential conflict of interest.

## Publisher's Note

All claims expressed in this article are solely those of the authors and do not necessarily represent those of their affiliated organizations, or those of the publisher, the editors and the reviewers. Any product that may be evaluated in this article, or claim that may be made by its manufacturer, is not guaranteed or endorsed by the publisher.
