# Supplementary material for: Humic Acid Modified by Being Incorporated Into Phosphate Fertilizer Increases Its Potency in Stimulating Maize Growth and Nutrient Absorption
Source: Front Plant Sci. 2022 May 19;13:885156. doi: 10.3389/fpls.2022.885156 (PMC9161291; doi:10.3389/fpls.2022.885156)
Supplement: Supplementary file 1 [file Data_Sheet_1.ZIP › Supplementary Material-Table S1.docx]

**Table S1** Interaction of humic acid type and their concentration on biomass, P, and N uptake of maize evaluated using two-factor analysis

|  | **Source** | **F Value** | ***Pr* > F** |  | **Type** | **Average value***  **(n = 12)** |  | **Concentration** | **Average value***  **(n = 6)** |
| --- | --- | --- | --- | --- | --- | --- | --- | --- | --- |
| Biomass | Type | 49.83 | <0.001 |  | HA | 4.487 a |  | 2.5 | 4.275 b |
|  | Concentration | 15.24 | <0.001 |  | PHA | 4.060 b |  | 5.0 | 4.603 a |
|  | Type × Concentration | 170.19 | <0.001 |  |  |  |  | 10 | 4.067 c |
|  |  |  |  |  |  |  |  | 25 | 4.148 bc |
| P uptake | Type | 310.03 | <0.001 |  | HA | 44.740 a |  | 2.5 | 37.840 c |
|  | Concentration | 60.62 | <0.001 |  | PHA | 37.066 b |  | 5.0 | 44.897 a |
|  | Type × Concentration | 308.67 | <0.001 |  |  |  |  | 10 | 38.343 c |
|  |  |  |  |  |  |  |  | 25 | 42.532 b |
| N uptake | Type | 55.10 | <0.001 |  | HA | 254.392 a |  | 2.5 | 245.877 a |
|  | Concentration | 11.54 | <0.001 |  | PHA | 228.718 b |  | 5.0 | 251.194 a |
|  | Type × Concentration | 118.89 | <0.001 |  |  |  |  | 10 | 224.446 b |
|  |  |  |  |  |  |  |  | 25 | 244.702 a |

Type indicates the type of humic acid, including HA and PHA. Concentration indicates the concentration of HA or PHA, including 2.5, 5, 10, and 25 mg C L^-1^. Means followed with the different lowercases within the same factor indicate a significant difference (*P* < 0.05). * Average value is the mean averaged across all treatments related to this factor (type or concentration).
