# Supplementary material for: Humic Acid Modified by Being Incorporated Into Phosphate Fertilizer Increases Its Potency in Stimulating Maize Growth and Nutrient Absorption
Source: Front Plant Sci. 2022 May 19;13:885156. doi: 10.3389/fpls.2022.885156 (PMC9161291; doi:10.3389/fpls.2022.885156)
Supplement: Supplementary file 1 [file Data_Sheet_1.ZIP › Supplementary Material-Figures.docx]

**Figure S1.** ^13^C NMR spectra and molecular weight distribution curve of HA **(A, C)** and PHA **(B, D)**

**Figure S2** Effects of 2.5-25 mg C L^-1^ HA and PHA on the **(A)** root P uptake, **(B)** shoot P uptake, **(C)** root N uptake, and **(D)** shoot N uptake. Error bars represent standard deviations (n = 3). Different lowercase letters above columns indicate significant differences between HA and control treatments, while different capital letters above columns indicate significant differences between PHA and control treatments at *P* < 0.05, as determined by LSD test. Significant differences between HA and PHA treatments at equivalent carbon concentrations were compared with two independent sample t-test, * *P* < 0.05; ** *P* < 0.01.
