# Supplementary figures and images for: Humic Acid Modified by Being Incorporated Into Phosphate Fertilizer Increases Its Potency in Stimulating Maize Growth and Nutrient Absorption
Source: Front Plant Sci. 2022 May 19;13:885156. doi: 10.3389/fpls.2022.885156 (PMC9161291; doi:10.3389/fpls.2022.885156)

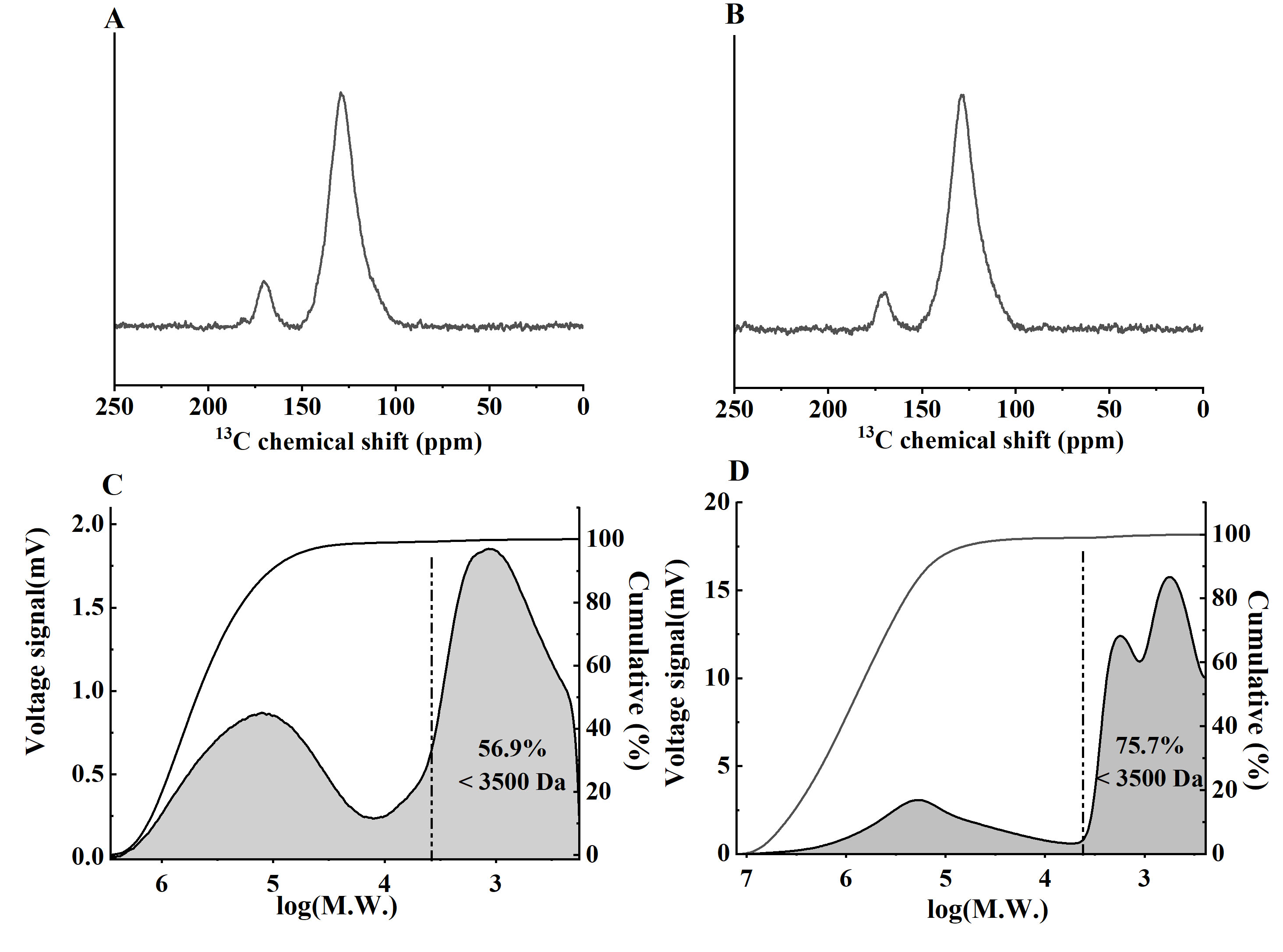

Supplement: Supplementary file 1 [file Data_Sheet_1.ZIP › Figure S1.jpg]

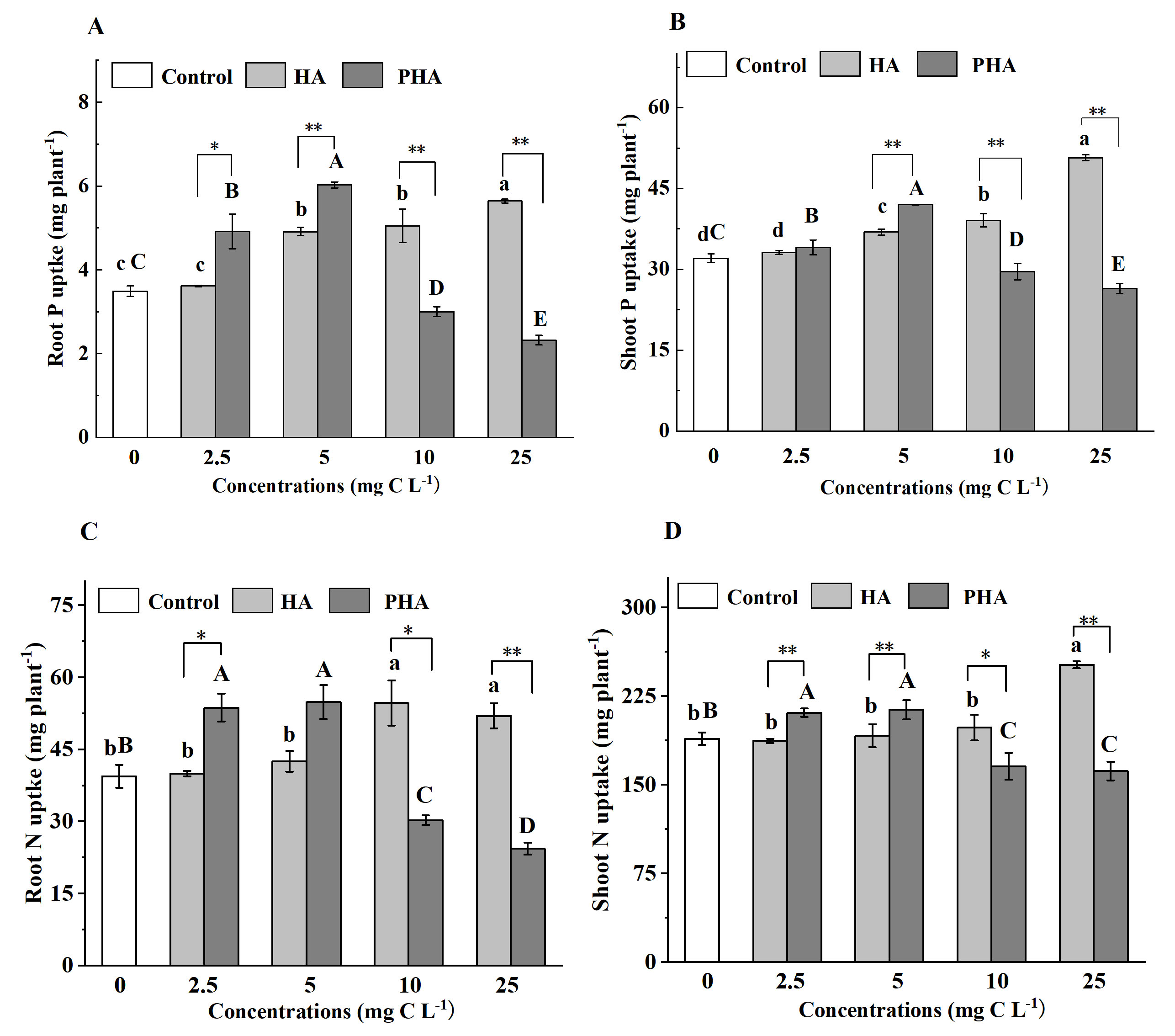

Supplement: Supplementary file 1 [file Data_Sheet_1.ZIP › Figure S2.jpg]
